# Comparison of supraglottic airway versus endotracheal intubation for the pre-hospital treatment of out-of-hospital cardiac arrest

**DOI:** 10.1186/cc10483

**Published:** 2011-10-10

**Authors:** Kentaro Kajino, Taku Iwami, Tetsuhisa Kitamura, Mohamud Daya, Marcus Eng Hock Ong, Tatsuya Nishiuchi, Yasuyuki Hayashi, Tomohiko Sakai, Takeshi Shimazu, Atsushi Hiraide, Masashi Kishi, Shigeru Yamayoshi

**Affiliations:** 1 Emergency and Critical Care Medical Center, Osaka Police Hospital, 10-31 Kitayama-cho Tennouji-ku, Osaka 543-0035, Japan; 2 Kyoto University, Health Services, Yoshida-Honmachi, Sakyo-ku, Kyoto 606-8501, Japan; 3 Department of Emergency Medicine, Oregon Health and Science University, 3181 SW Sam Jackson Park Road, mail code CR-114, Portland, OR 97239-3098, USA; 4 Department of Emergency Medicine, Singapore General Hospital, Outram Road, Singapore 169608, Singapore; 5 Department of Critical Care and Emergency Medicine, Osaka City University Graduate School of Medicine, 1-5-17 Asahimachi, Abeno-ku, Osaka 545-8585, Japan; 6 Senri Critical Care Medical Center, Osaka Saiseikai Senri Hospital, 1-1 D5, Tsukumodai, Suita, Osaka 565-0862, Japan; 7 Department of Trauma and Critical Care Medicine and Burn Centers, Social Insurance Chukyo Hospital, 1-1-10 Sanjyo Minami-ku, Nagoya, Aichi 457-8510, Japan; 8 Department of Traumatology and Acute Critical Medicine, Osaka University Graduate School of medicine, 2-15 Yamada-Oka, Suita City, Osaka 565-0871, Japan; 9 ER Medicine, Kinki University Faculty of Medicine, 377-2 Ouno higashi Osaka-Sayama, Osaka 589-8511, Japan

**Keywords:** cardiac arrest, endotracheal intubation, Supraglottic airway, advanced airway, pre-hospital, resuscitation

## Abstract

**Introduction:**

Both supraglottic airway devices (SGA) and endotracheal intubation (ETI) have been used by emergency life-saving technicians (ELST) in Japan to treat out-of-hospital cardiac arrests (OHCAs). Despite traditional emphasis on airway management during cardiac arrest, its impact on survival from OHCA and time dependent effectiveness remains unclear.

**Methods:**

All adults with witnessed, non-traumatic OHCA, from 1 January 2005 to 31 December 2008, treated by the emergency medical services (EMS) with an advanced airway in Osaka, Japan were studied in a prospective Utstein-style population cohort database. The primary outcome measure was one-month survival with neurologically favorable outcome. The association between type of advanced airway (ETI/SGA), timing of device placement and neurological outcome was assessed by multiple logistic regression.

**Results:**

Of 7,517 witnessed non-traumatic OHCAs, 5,377 cases were treated with advanced airways. Of these, 1,679 were ETI while 3,698 were SGA. Favorable neurological outcome was similar between ETI and SGA (3.6% versus 3.6%, *P *= 0.95). The time interval from collapse to ETI placement was significantly longer than for SGA (17.2 minutes versus 15.8 minutes, *P *< 0.001). From multivariate analysis, early placement of an advanced airway was significantly associated with better neurological outcome (Adjusted Odds Ratio (AOR) for one minute delay, 0.91, 95% confidence interval (CI) 0.88 to 0.95). ETI was not a significant predictor (AOR 0.71, 95% CI 0.39 to 1.30) but the presence of an ETI certified ELST (AOR, 1.86, 95% CI 1.04 to 3.34) was a significant predictor for favorable neurological outcome.

**Conclusions:**

There was no difference in neurologically favorable outcome from witnessed OHCA for ETI versus SGA. Early airway management with advanced airway regardless of type and rhythm was associated with improved outcomes.

## Introduction

### Background

Sudden cardiac arrest is a major public health problem in the industrialized world [1]. Endotracheal intubation (ETI) has been considered the 'gold standard' for airway management during cardiopulmonary arrest in western countries. However, the training and maintenance of ETI skills for emergency medical services (EMS) personnel is expensive and it is not clear whether ETI contributes to improved outcome from out-of-hospital cardiac arrest (OHCA) compared to other available methods of airway management [2]. Recently, there has been an increased interest in the EMS use of supraglottic airways (SGA) during resuscitation since they can be placed quickly and with minimal training.

In Japan, advanced airway devices (Combitube, Laryngeal mask airway and Laryngeal Tube) have been used by Emergency Life-Saving Technicians (ELSTs) since 1991, and specially-trained ELSTs have been permitted to use ETI since 2004 [3]. The use of advanced airway devices in Japan, including ETI for OHCA patients, has increased year by year [4]. However, it is still unclear whether survival from OHCA is improved by the use of ETI instead of the SGA.

Equally unclear is when an advanced airway device should be placed during a resuscitation attempt and the introduction of advanced airway skills to EMS for OHCA has not been associated with beneficial effects [5-7]. While the efficacy of advanced airway management has been demonstrated in selected studies [8,9], little is known regarding the time-dependent effectiveness of advanced airway management. Of note, several EMS systems have trained personnel to delay ETI placement until at least three to four minutes of chest compressions have been completed [10].

We conducted a large-scale, observational population-based cohort study of patients who experienced adult-witnessed, non-traumatic OHCA and were treated by EMS personnel using advanced airways (ETI or SGA) in Osaka Prefecture, Japan. In this study, we compared the difference of outcomes from OHCA between ETI and SGA. In addition, we evaluated the association between outcomes and the time-course from collapse to advanced airway placement.

## Materials and methods

### Study design and setting

This study was an observational, population-based cohort study that used a prospective, Utstein-Style population cohort database. The study was conducted in Osaka Prefecture, Japan at all hospitals that receive emergency OHCA cases from the prefectures' fire-station-based ambulances.

Cardiac arrest was defined as the cessation of cardiac mechanical activity as confirmed by the absence of signs of circulation. The arrests were classified into those of presumed cardiac and non-cardiac origin, the latter of which were caused by external causes, including trauma, hanging, drowning, drug overdose, asphyxia, respiratory diseases, cerebrovascular diseases, malignant tumors and any other non-cardiac causes. These diagnoses were made by the physician in charge, in collaboration with EMS providers. Cardiac etiology and non-cardiac etiology without trauma were defined as non-traumatic causes [11-13].

The research protocol, including a privacy policy, was approved by the institutional review board of Osaka University, the organizer of this project, with the assent of the EMS authorities and local governments in the prefecture. The requirement of written informed consent was waived.

### The EMS system in Osaka

Osaka Prefecture has 8.8 million residents in a 1,892 km^2 ^area of both urban and rural communities. It has 34 fire stations with a corresponding number of emergency dispatch centers. Pre-hospital life support is provided 24 hours each day by a fire-station-based EMS system, which is single-tiered in 32 stations and two-tiered in 2 stations.

Each fire ambulance has three EMS personnel with at least one ELST. ELSTs are authorized to use an automated external defibrillator, to insert an intravenous line, and to place advanced airway management devices for OHCA patients under on-line medical control direction. In Japan, EMS personnel are not permitted to terminate resuscitation in the field and all patients on whom resuscitation is attempted are transported to the hospital. Until September 2006, all EMS providers performed cardiopulmonary resuscitation (CPR) according to the Japanese Guidelines based on the American Heart Association, European Resuscitation Council, and the International Liaison Committee on Resuscitation 2000 Guidelines using a 15:2 compression-to-ventilation ratio. After September 2006, they switched to a ratio of 30:2 based on the 2005 Guidelines [14]. Public-access defibrillation programs have been promoted in Japan since July 2004. SGA devices have been used in Japan since the ELST system started in 1991. Beginning in 2004, specially-trained ELSTs have been permitted to use ETI under on-line medical control direction. (Not all ELSTs are so trained.) When ELSTs decided to use an advanced airway for eligible OHCA patients, standard ELSTs can use only SGA on the scene whereas the choice of either ETI or SGA by specially-trained ELSTs was at each ELST's discretion.

### The requirements for becoming an ELST

There are two processes to becoming an ELST. The first is through the education system in the fire department. To become an Emergency Medical Technician (EMT), fire department officers have to receive fundamental medical education for 250 hours in the fire academy. After being engaged in life-saving at the pre-hospital settings as an EMT for more than 5 years or 2,000 hours, EMTs must pass the national examination of ELST after receiving professional medical education and training at the fire academy for at least one year.

The second way is through the education system in the EMT school and college. To become an ELST, candidates must pass the national examination of ELST after receiving professional medical education and training about life-saving at the certified EMT school or college for at least two years.

### Process of the endotracheal intubation authorization in Japan

Subject: Emergency life saving technicians

Authorizing organization: Regional medical control committee

Training periods: More than 62 periods (1 period = 50 minutes)

Practical training: More than 30 successful intubations for patients in the hospital's operating room under the attending physician.

### Protocol for supraglottic airways placement

If there are indications for authorized action (indication: the cause of death, patient's status, the distance to the receiving hospital, and so on) and ELST is permitted by on-line medical control, the ELST is able to do SGA placement. Placement time is within 10 seconds/attempt. There are no restrictions on the number of attempts. After SGA placement, non-synchronized CPR is possible.

### Protocol for endotracheal intubation

The protocol for endotracheal intubation of cardiac arrest patients who have both lack of pulse and apnea is as follows. 1. The patient meets the indications for endotracheal intubation. 2. It is impossible to maintain the patient's airway with SGA, because of (1) asphyxia due to foreign-body airway obstruction, (2) or in cases in which the medical control doctor judges ETI to be required. 3. Patients are ineligible for endotracheal intubation, because there is: (1) suspected cervical spine injury, (2) head-tilt difficulty, (3) trismus, (4) difficulty with laryngoscope insertion, (5) difficulty with larynx expansion after laryngoscope insertion, (6) difficulty in visualizing the vocal chords, (7) prolonged unsuccessful attempts, (8) the ELST on the scene is not certified to perform endotracheal intubation, and (9) rapid sequence intubation (paralysis and sedation) is not used for ETI as it is only indicated for cardiac arrest.

### Selection of participants

From 1 January 2005 through 31 December 2008, this study enrolled all persons in Osaka Prefecture, Japan, aged 18 years or older who suffered from adult-witnessed non-traumatic OHCA, and who were treated with an advanced airway by ELSTs. Participants were enrolled from a prospective Utstein-Style population cohort database.

### Data collection and processing

Data were prospectively collected using a data collection tool designed by the project steering committee. Included were all core data elements recommended in the Utstein style for OHCA [11,12], including age, gender, etiology, first documented rhythm, resuscitation time-course, bystander-initiated CPR, location, ELST status, final device type of advanced airway, epinephrine administration, return of spontaneous circulation (ROSC), hospital admission, one-month survival and neurological status at one month after the event. The data sheet was filled out by the EMS personnel in cooperation with the physicians in charge of the patient. It was then transferred to the Information Center for Emergency Medical Services of Osaka and reviewed by the investigators. If the information provided on the data sheet was unclear or incomplete, it was returned to the appropriate EMS personnel for completion. All survivors were followed for up to one month after the event, and the neurological outcomes were obtained by the responsible EMS personnel with the cooperation of the Osaka Medical Association and medical institutions in this area. Neurological status was determined using the Cerebral Performance Categories [11,14].

### Methods of measurement

The primary predictor variable was the presence of an advanced airway (ETI versus SGA). The secondary predictor variable was time-course from collapse to advanced airway placement. The primary study outcome measure was neurologically favorable one-month survival, defined as a Cerebral Performance Category score of 1 or 2. Secondary outcome measures were ROSC, admission to hospital, and one-month survival. ROSC was defined as the restoration of a sustained spontaneous perfusing rhythm [11,12].

### Primary data analysis

Outcomes were evaluated based on whether OHCA patients received ETI or SGA. We used analysis of *t*-test for continuous variables and chi-squared tests for categorical variables. Logistic regression analysis was used to determine the survival association of each predictor, and odds ratios (ORs) and their 95% confidence intervals (CIs) were calculated, controlling for age, gender, first recorded cardiac rhythm, time course of resuscitation, presence of bystander CPR, arrest location, ELST status, presence of ETI, epinephrine administration and etiology. Next, eligible participants were divided into quartiles based on the time from collapse to advanced airway placement (groups Q1 to Q4; Q1: ≤10 minutes, Q2: 11 to 14 minutes, Q3: 15 to 19 minutes, Q4: ≥20 minutes). We compared outcomes of the Q2 to Q4 groups with those of the Q1 group. In addition, we evaluated the outcomes according to the initial cardiac rhythm. Statistical analyses were performed using the SPSS statistical package version 15.0J (SPSS, Inc., Chicago, IL, USA). A *P*-value < 0.05 was considered statistically significant.

## Results

### Overview of OHCA patients in Osaka

**Figure 1:**
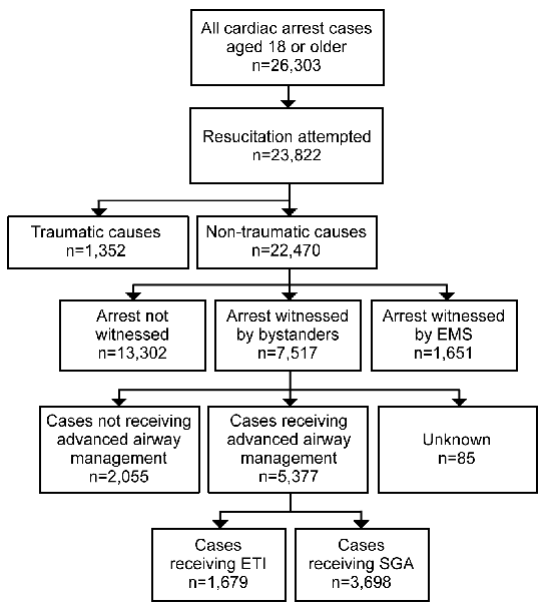
**Out-of-hospital cardiac arrest cases for analysis**. EMS, emergency medical services; SGA, supraglottic airway devices; ETI, endotracheal intubation.

Figure 1 provides an overview of all OHCA cases during the four-year study period. A total of 26,303 adult OHCAs were documented and resuscitation was attempted for 23,822 patients. Of these, 22,470 resuscitated patients were non-traumatic, and 7,517 of these were OHCA patients witnessed by adult bystanders. Of this number, 5,377 eligible participants were treated with an advanced airway by ELSTs; 1,679 with ETI (ETI group) and 3,698 with the SGA (SGA group).

### Participant and EMS characteristics, and treatment outcomes, by the type of advanced airway placed

Table 1 shows the characteristics of eligible participants by the type of advanced airway placed. The ETI group had a greater mean age than did the SGA group (73.8 years versus 71.9 years, *P *< 0.001). The proportion of participants who received epinephrine at the scene was significantly higher in the ETI group than in the SGA group (27.1% versus 5.9%, *P *< 0.001). The intervention time (from collapse to advanced airway placement) and the total activity time (from collapse to hospital arrival) was significantly longer in the ETI group compared to the SGA group (17.2 minutes versus 15.8 minutes, *P *< 0.001, and 33.9 minutes versus 30.3 minutes *P *< 0.001, respectively).

**Table 1:** Participant and EMS characteristics among participants with out-of-hospital cardiac arrest who received advanced airway management.

Characteristics	Total *n *= 5,377	ETI group *n *= 1,679	SGA group *n *= 3,698	*P*-value
	**n (%)**	**n (%)**	**n (%)**	
Men	3,312 (61.6)	1,021 (60.8)	2,291 (62.0)	0.425
Bystander CPR	2,158 (40.1)	686 (40.9)	1,472 (39.8)	0.466
Public location	694 (12.9)	208 (12.4)	486 (13.1)	0.445
Ventricular fibrillation	900 (16.8)	278 (16.6)	622 (16.9)	0.796
Epinephrine	674 (12.5)	455 (27.1)	219 (5.9)	**< 0.001**
	**Mean ± SD**	**Mean ± SD**	**Mean ± SD**	
Age (years)	72.5 ± 15.0	73.8 ± 14.6	71.9 ± 15.2	**< 0.001**
EMS care interval (minutes)				
Collapse to EMS arrival	9.29 ± 6.90	9.17 ± 6.81	9.35 ± 6.94	0.369
Collapse to EMS CPR	10.87 ± 7.00	10.80 ± 7.01	10.90 ± 6.99	0.615
Collapse to airway placement	16.22 ± 7.54	17.17 ± 7.50	15.79 ± 7.52	**< 0.001**
Collapse to hospital arrival	31.41 ± 9.29	33.90 ± 9.16	30.28 ± 9.12	**< 0.001**

CPR, cardiopulmonary resuscitation; EMS, emergency medical service; ETI,: endotracheal intubation; SGA, supraglottic airways

Treatment outcomes by the type of advanced airway used are presented in Table 2. The proportion of pre-hospital ROSC was significantly higher in the ETI group than in the SGA group (16.6% versus 10.1%, *P *< 0.001), as was the proportion of ROSC in the emergency department (47.8% versus 44.4%, *P *= 0.002). However, one-month survival with favorable neurological outcome was not different between the ETI and SGA groups.

**Table 2:** Outcomes for out-of-hospital cardiac arrest by the type of airway management device

Outcome	Total n = 5,377	ETI n = 1,679	SGA n = 3,698	*P*-value
	n (%)	n (%)	n (%)	
Pre-hospital ROSC	654 (12.2)	279 (16.6)	375 (10.1)	**< 0.001**
ROSC in ED	2,445 (45.5)	802 (47.8)	1,643 (44.4)	**0.002**
Hospital admission	2,100 (39.1)	688 (41.0)	1,412 (38.2)	0.052
One-month survival	541 (10.1)	180 (10.7)	361 (9.8)	0.279
Neurologically favorable outcome	194 (3.6)	61 (3.6)	133 (3.6)	0.945

ED, emergency department; ETI, endotracheal intubation; ROSC, return of spontaneous circulation; SGA, supraglottic airway

### Participant and EMS characteristics associated with neurologically favorable outcome

**Figure 2:**
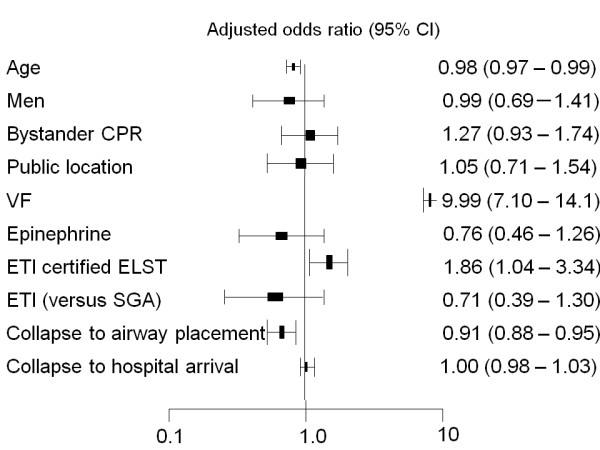
**Multivariate-adjusted odds ratios for neurologically favorable survival**. SGA, supraglottic airway devices; ETI, endotracheal intubation; ELST, emergency Life-Saving Technicians; CPR, cardiopulmonary resuscitation; VF, ventricular fibrillation.

Figure 2 shows the participant and EMS characteristics associated with favorable neurological outcome after OHCA. After adjusting for confounding variables, ETI (versus SGA) was not a significant predictor of a favorable outcome. The time to advanced airway placement (in one minute increments) and the presence of an ETI-certified ELST were significant predictors of a favorable outcome (adjusted OR, 0.91; 95% CI 0.88 to 0.95; *P *< 0.011, adjusted OR, 1.86; 95% CI 1.04 to 3.34, *P *< 0.01; respectively).

### Time of advanced airway placement (by quartile groups) and favorable neurological outcome

**Figure 3:**
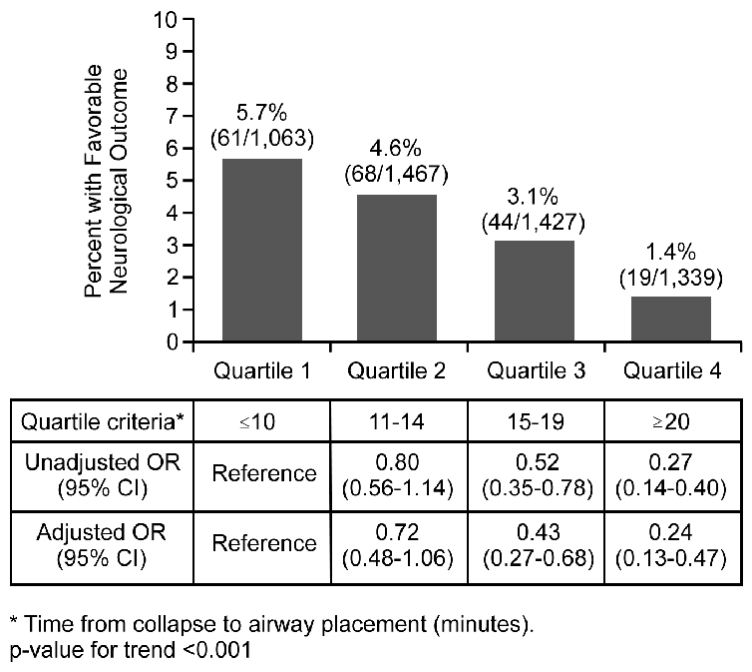
**Odds ratios of favorable neurological outcome by quartile time of advanced airway management**. OR, odds ratio; CIs, confidence intervals.

One-month survival with favorable neurological outcome by quartile group is illustrated in Figure 3. The proportion of favorable neurological outcome among OHCA patients with advanced airway management decreased as time-to-placement increased: 5.7% in Q1, 4.6% in Q2, 3.1% in Q3, and 1.4% in Q4. In subgroup analyses (Table 3), we evaluated the time-dependence of neurologically favorable outcome according to the initial cardiac rhythm, there was, however, no rhythm variability in the outcome.

**Table 3:** Odds ratios of favorable neurological outcome by quartile time of advanced airway management according to initial cardiac rhythm.

Quartile criteria	Quartile 1	Quartile 2	Quartile 3	Quartile 4	*P*-value
	≤10	11 to 14	15 to 19	≥20	for trend*
**VF/pulseless VT**					
Neurologically favorable outcome, % (n/N)	23.4 (43/184)	17.9 (47/263)	10.8 (30/277)	7.2 (12/167)	
Unadjusted OR	Reference	0.71	0.4	0.25	< 0.001
(95% CI)		(0.45 to 1.14)	(0.24 to 0.66)	(0.13 to 0.50)	
Adjusted OR	Reference	0.65	0.37	0.22	< 0.001
(95% CI)		(0.40 to 1.06)	(0.21 to 0.66)	(0.10 to 0.50)	
**PEA/Asystole**					
Neurologically favorable outcome, % (n/N)	2.1 (18/877)	1.7 (21/1,202)	1.2 (14/1,148)	0.6 (7/1,170)	
Unadjusted OR	Reference	0.85	0.59	0.29	< 0.001
(95% CI)		(0.45 to 1.60)	(0.29 to 1.19)	(0.12 to 0.69)	
Adjusted OR	Reference	0.82	0.57	0.27	< 0.001
(95% CI)		(0.41 to 1.61)	(0.26 to 1.26)	(0.09 to 0.81)	

VF, ventricular fibrillation; VT, ventricular tachycardia; PEA, pulseless electrical activity; OR, odds ratio; CI, confidence interval

* Time from collapse to airway placement (minutes)

## Discussion

From this large prospective population-based registry of OHCA, we investigated not only the difference in outcomes between ETI and SGA airway devices but also the time-dependent effectiveness of airway management for non-traumatic OHCA. The device used was not a significant predictor for favorable neurological outcome after OHCA. The results do suggest that rapid advanced airway insertion-regardless of device and rhythm-and ETI certification for ELSTs contribute to improved outcomes after OHCA.

Although some studies have examined the success rate or the complication rates associated with airway management [15], few have compared OHCA outcomes with different airway devices. Rabitisch *et al*. [16] found no significant difference in outcomes between ETI and SGA that were placed by emergency physicians in the out-of-hospital setting. Cady *et al*. [17] found no significant difference in survival to hospital discharge between Emergency Medical Technician-Basic, Esophageal Tracheal Combitube management and paramedic ETI. Our finding that OHCA outcomes were not significantly different between the ETI and SGA devices is consistent with the findings of these previous studies.

Why have studies shown no benefit on survival from OHCA with ETI compared to the SGA? Insertion of ETI might require more interruption of chest compressions than does SGA. According to Wang *et al*. [18], the median total duration of all endotracheal intubation-associated CPR interruptions was 109.5 seconds. Delayed treatments among OHCA patients in pre-hospital settings have been associated with poorer outcomes [19]. Further studies to investigate characteristics and outcomes following ETI are warranted. There is a need for a randomized controlled trial of different airway placement strategies in OHCA.

The present study underscored the importance of early advanced airway placement regardless of device type. Our findings were consistent with those of Iwami *et al*. [20], who previously reported that early advanced airway placement is associated with improved outcomes after OHCA, and with those of Shy *et al*. [8] who reported similar results. Rapid advanced airway placement is, therefore, a vital factor to be considered for resuscitation of OHCA patients at the scene, along with minimum interruption of chest compressions and early defibrillation [8,9].

The present study also showed that ETI certification for attending ELSTs contributed to improved outcomes after OHCA, which suggests that the additional experience and training received by ELSTs may be very important in improving outcomes. Studies have shown that the procedural experience of rescuers is associated with the improved survival of patients with and without cardiac arrest after out-of-hospital tracheal intubation [21,22]. ETI use by EMS personnel is relatively new in Japan, being permitted since 2004. Therefore, EMS systems in Japan need to increase the ETI experience of EMS personnel. To improve outcomes with advanced airway management by ELSTs in Japan, concentrated training in hospital operating rooms (currently done) and in the emergency room (not currently done) is needed to increase the quality and experience of ETI by ELSTs.

This observational study has several important limitations. First, we have no information as to why the ELSTs chose a particular type of advanced airway device. Although the criteria and sequence for ETI use is specified in the EMS protocol, it appears that the protocol was not always followed for OHCA patients with presenting rhythms that required early defibrillation. This suggests that selection bias by the EMS providers might account for some of the differences observed in this study. Second, this was not a randomized controlled trial and although we adjusted for confounding factors in the multivariable analysis, other unknown confounding factors might exist which could have affected our results. Third, we did not obtain data on the CPR quality (compression rate, compression depth, CPR fraction and ventilation rate) of the EMS providers and did not monitor EMS CPR process data. However, the EMS system was generally uniform in this study area [14] and it is unlikely that a difference in CPR quality would account for the differences between the two groups. Fourth, we have no data on the quality of advanced airway management by the individual ELST. It is possible that the ELST's performance status (intubation frequency, chest compression interruption periods, and intubation success rate) may have influenced the outcomes after OHCA [21,22]. Fifth, information on the new CPR guidelines during the study period might affect the relationship between advanced airway management and the outcome. Sixth, as with all multi-site epidemiological studies, data integrity, validity and ascertainment bias are potential limitations. The uniform data collection, consistent definitions, time synchronization process and large sample size in this population-based cohort study were intended to minimize these potential sources of bias.

## Conclusions

Despite a longer time interval for collapse to airway placement for ETI compared to SGA, the devices are equally effective for on-site out-of-hospital airway management after OHCA. In patients who received an advanced airway, early advanced airway placement -- regardless of device and rhythm -- is associated with improved outcomes in OCHA patients, as is ETI certification for attending ELSTs.

## Key messages

• The intervention time (from collapse to advanced airway placement) was significantly longer in the ETI group compared to the SGA group (17.2 minutes versus 15.8 minutes, *P *< 0.001).

• One-month survival with favorable neurological outcome was not different between the ETI and SGA groups (3.6% versus 3.6%, *P *= 0.945).

• The presence of an ETI-certified ELST was a significant predictor of a favorable outcome (adjusted OR, 0.91; 95% CI 0.88 to 0.95; *P *< 0.011, adjusted OR, 1.86; 95% CI 1.04 to 3.34, *P *< 0.01; respectively).

• The proportion of favorable neurological outcomes among OHCA patients with advanced airway management decreased as time-to-placement increased: 5.7% in Q1, 4.6% in Q2, 3.1% in Q3, and 1.4% in Q4. (Q1: ≤10 minutes, Q2: 11 to 14 minutes, Q3: 15 to 19 minutes, Q4: ≥20 minutes).

• In patients who received an advanced airway, early advanced airway placement-regardless of device and rhythm is associated with improved outcomes in OCHA patients, as is ETI certification for attending ELSTs.

## Abbreviations

AOR: adjusted odds ratio; CIs: confidence intervals; CPR: cardiopulmonary resuscitation; ELST: Emergency Life-Saving Technicians; EMS: Emergency medical services; ETI: endotracheal intubation; OHCAs: out-of-hospital cardiac arrests; OR: odds ratio; ROSC: return of spontaneous circulation; SGA: supraglottic airway devices; VF: ventricular fibrillation.

## Competing interests

The authors declare that they have no competing interests.

## Authors' contributions

KK, TI, TK, MD, MO, TN, YH, TS, TS, AH, MK and SY participated in the idea formation, study design, data analyses, interpretation of results and writing of the report. All the authors read and approved the final manuscript.

## References

[B1] 2005 American Heart Association Guidelines for Cardiopulmonary Resuscitation and Emergency Cardiovascular Care. Part 7.1: Adjuncts for airway control and ventilation Circulation 2005 112 IV-51 IV-57 10.1161/CIRCULATIONAHA.105.166550 16314375

[B2] Nolan JP Lockey D Airway management for out-of-hospital cardiac arrest--more data required Resuscitation 2009 80 1333 1334 10.1016/j.resuscitation.2009.11.001 19942081

[B3] Tanigawa K Shigematsu A Choice of airway devices for 12,020 cases of nontraumatic cardiac arrest in Japan Prehosp Emerg Care 1998 2 96 100 10.1080/10903129808958850 9709326

[B4] Ambulance Service Planning Office of Fire and Disaster Management Agency of Japan Effect of first aid for cardiopulmonary arrest http://www.fdma.go.jp/neuter/topics/houdou/2101/210122-1houdou.pdf (in Japanese)

[B5] Gausche M Lewis RJ Stratton SJ Haynes BE Gunter CS Goodrich SM Poore PD McCollough MD Henderson DP Pratt FD Seidel JS Effect of out-of-hospital pediatric endotracheal intubation on survival and neurological outcome: a controlled clinical trial JAMA 2000 283 783 790 10.1001/jama.283.6.783 10683058

[B6] Stiell IG Wells GA Field B Spaite DW Nesbitt LP De Maio VJ Nichol G Cousineau D Blackburn J Munkley D Luinstra-Toohey L Campeau T Dagnone E Lyver M Advanced cardiac life support in out-of-hospital cardiac arrest N Engl J Med 2004 351 647 656 10.1056/NEJMoa040325 15306666

[B7] Lecky F Bryden D Little R Tong N Moulton C Emergency intubation for acutely ill and injured patients Cochrane Database Syst Rev 2008 CD001429 18425873 10.1002/14651858.CD001429.pub2 PMC7045728

[B8] Shy BD Rea TD Becker LJ Eisenberg MS Time to intubation and survival in prehospital cardiac arrest Prehosp Emerg Care 2004 8 394 399 15626000 10.1016/j.prehos.2004.06.013

[B9] Wong ML Carey S Mader TJ Wang HE Time to invasive airway management and resuscitation outcomes after inhospital cardiopulmonary arrest Resuscitation 2010 81 182 186 10.1016/j.resuscitation.2009.10.027 20022157 PMC3068860

[B10] Garza AG Gratton MC Salomone JA Lindholm D McElroy J Archer R Improved patient survival using a modified resuscitation protocol for out-of-hospital cardiac arrest Circulation 2009 119 2597 2605 10.1161/CIRCULATIONAHA.108.815621 19414637

[B11] Jacobs I Nadkarni V Bahr J Berg RA Billi JE Bossaert L Cassan P Coovadia A D'Este K Finn J Halperin H Handley A Herlitz J Hickey R Idris A Kloeck W Larkin GL Mancini ME Mason P Mears G Monsieurs K Montgomery W Morley P Nichol G Nolan J Okada K Perlman J Shuster M Steen PA Sterz F Cardiac arrest and cardiopulmonary resuscitation outcome reports: update and simplification of the Utstein templates for resuscitation registries: a statement for healthcare professionals from a task force of the International Liaison Committee on Resuscitation (American Heart Association, European Resuscitation Council, Australian Resuscitation Council, New Zealand Resuscitation Council, Heart and Stroke Foundation of Canada, InterAmerican Heart Foundation, Resuscitation Councils of Southern Africa) Circulation 2004 110 3385 3397 10.1161/01.CIR.0000147236.85306.15 15557386

[B12] Cummins RO Chamberlain DA Abramson NS Allen M Baskett PJ Becker L Bossaert L Delooz HH Dick WF Eisenberg MS Recommended guidelines for uniform reporting of data from out-of-hospital cardiac arrest: the Utstein Style. A statement for health professionals from a task force of the American Heart Association, the European Resuscitation Council, the Heart and Stroke Foundation of Canada, and the Australian Resuscitation Council Circulation 1991 84 960 975 1860248 10.1161/01.cir.84.2.960

[B13] Kitamura T Iwami T Kawamura T Nagao K Tanaka H Hiraide A Bystander-initiated rescue breathing for out-of-hospital cardiac arrests of noncardiac origin Circulation 2010 122 293 299 10.1161/CIRCULATIONAHA.109.926816 20606122

[B14] Kajino K Iwami T Daya M Nishiuchi T Hayashi Y Kitamura T Irisawa T Sakai T Kuwagata Y Hiraide A Kishi M Yamayoshi S Impact of transport to critical care medical centers on outcomes after out-of-hospital cardiac arrest Resuscitation 2010 81 549 554 10.1016/j.resuscitation.2010.02.008 20303640

[B15] Wang HE Mann NC Mears G Jacobson K Yealy DM Out-of-hospital airway management in the United States Resuscitation 2011 82 378 385 10.1016/j.resuscitation.2010.12.014 21288624

[B16] Rabitsch W Schellongowski P Staudinger T Hofbauer R Dufek V Eder B Raab H Thell R Schuster E Frass M Comparison of a conventional tracheal airway with the Combitube in an urban emergency medical services system run by physicians Resuscitation 2003 57 27 32 10.1016/S0300-9572(02)00435-5 12668296

[B17] Cady CE Weaver MD Pirrallo RG Wang HE Effect of emergency medical technician-placed Combitubes on outcomes after out-of-hospital cardiopulmonary arrest Prehosp Emerg Care 2009 13 495 499 10.1080/10903120903144874 19731162

[B18] Wang HE Simeone SJ Weaver MD Callaway CW Interruptions in cardiopulmonary resuscitation from paramedic endotracheal intubation Ann Emerg Med 2009 54 645 652 10.1016/j.annemergmed.2009.05.024 19573949

[B19] Gold LS Fahrenbruch CE Rea TD Eisenberg MS The relationship between time to arrival of emergency medical services (EMS) and survival from out-of-hospital ventricular fibrillation cardiac arrest Resuscitation 2010 81 622 625 10.1016/j.resuscitation.2010.02.004 20207470

[B20] Iwami T Nichol G Hiraide A Hayashi Y Nishiuchi T Kajino K Morita H Yukioka H Ikeuchi H Sugimoto H Nonogi H Kawamura T Continuous improvements of chain of survival increased survival after out-of-hospital cardiac arrests: a large-scale population-based study Circulation 2009 119 728 734 10.1161/CIRCULATIONAHA.108.802058 19171854

[B21] Wang HE Balasubramani GK Cook LJ Lave JR Yealy DM Out-of-hospital endotracheal intubation experience and patient outcomes Ann Emerg Med 2010 55 527 537 10.1016/j.annemergmed.2009.12.020 20138400 PMC3071147

[B22] Wang HE Abo BN Lave JR Yealy DM How would minimum experience standards affect the distribution of out-of-hospital endotracheal intubations? Ann Emerg Med 2007 50 246 252 10.1016/j.annemergmed.2007.04.023 17597255

